# Information Diffusion on Social Media During Natural Disasters

**DOI:** 10.1109/TCSS.2017.2786545

**Published:** 2018-01-11

**Authors:** Rongsheng Dong, Libing Li, Qingpeng Zhang, Guoyong Cai

**Affiliations:** 1 Guangxi Key Laboratory of Trusted SoftwareGuilin University of Electronic TechnologyGuilin541004China; 2 Mintaian Insurance Surveyors & Loss Adjusters Group Company Ltd.Shenzhen518000China; 3 Department of Systems Engineering and Engineering ManagementCity University of Hong KongHong Kong; 4 Shenzhen Research Institute of City University of Hong KongShenzhen518000China

**Keywords:** Emergency response, online collective behavior, Sina-Weibo, social network analysis

## Abstract

Social media analytics has drawn new quantitative insights of human activity patterns. Many applications of social media analytics, from pandemic prediction to earthquake response, require an in-depth understanding of how these patterns change when human encounter unfamiliar conditions. In this paper, we select two earthquakes in China as the social context in Sina-Weibo (or Weibo for short), the largest Chinese microblog site. After proposing a formalized Weibo information flow model to represent the information spread on Weibo, we study the information spread from three main perspectives: individual characteristics, the types of social relationships between interactive participants, and the topology of real interaction networks. The quantitative analyses draw the following conclusions. First, the shadow of Dunbar’s number is evident in the “declared friends/followers” distributions, and the number of each participant’s friends/followers who also participated in the earthquake information dissemination show the typical power-law distribution, indicating a rich-gets-richer phenomenon. Second, an individual’s number of followers is the most critical factor in user influence. Strangers are very important forces for disseminating real-time news after an earthquake. Third, two types of real interaction networks share the scale-free and small-world property, but with a looser organizational structure. In addition, correlations between different influence groups indicate that when compared with other online social media, the discussion on Weibo is mainly dominated and influenced by verified users.

## I. Introduction

The 2009 DARPA Red Balloon Challenge offered the Internet and social networking a chance to demonstrate their vast potential to solve a distributed, time-critical, highly distributed public problem [1], [2]. And the modern social network study has inferred much new quantitative knowledge about human activity patterns, such as influencer’s identification [2]–[5], the network topology measurement [6], [7], trust analysis [8]–[10], social hot spot-tracing [11]–[13], and the dynamics of information spread [14]–[16]. However, many applications, from pandemic prediction to earthquake response, require an understanding of how these patterns change when human encounter unfamiliar conditions [17], [18]. Especially for China, who suffered from frequent natural disasters, the understanding of how the behaviors of hundreds of millions of Web users change is very important. The empirical study of the human flesh search (HFS) [19], [20], for one, provided quantitative insights into these collective responses of Web users in China. Inspired by previous research on Web users collective responses, we choose two empirical cases in China-Yi’liang (2012) and Ya’an earthquakes (2013). What makes it additionally useful is that many densely populated areas in mainland China, such as Sichuan, Fujian, and so on, are frequent earthquake areas and often suffered severe damage from earthquakes.

At present for earthquake topics, there are two main types of studies: detecting seismic waves and enhancing rescue efforts. The former focuses on how to improve the accuracy of magnitude of earthquake forecasting or issue warnings as early as possible [21]–[24], such as the Did You Feel It system. And the later studies [25]–[27] try to explore ideas to cope with earthquake relief, postearthquake reconstruction, and to improve the mental health status of rescuers. This paper focuses on the latter effort from a social network perspective, with a particular focus on information diffusion and social networking behaviors.

In social network study, the above-mentioned focus [25]–[27] can be seen as a nonlinear superposition of a multitude of social interaction networks, where nodes represent individuals and edges capture a variety of different social relations. However, after further study, a group of researchers represented by Huberman *et al.* [28] found that social interactions within Twitter cannot be inferred directly from a declared relationship set of friends and followers: many users interact with few other people in their declared relationship network [28]–[31]. The key problem is that the structure of the underlying interaction network is not visible and must be inferred from the flow of information between individuals, which poses a serious challenge to our efforts to understand how the structure of the network affects social dynamics and the spread of information [32].

**Fig. 1.**
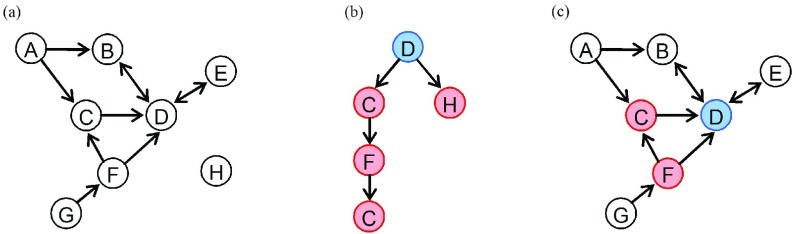
Typical multirelational participant network on Twitter/Weibo. (a) Declared social network, where nodes represent users and directed edges represent relationships of followers or friends. (b) Information cascade of one tweet, where nodes represent participating users and directed edges indicate tweets citing relationships. (c) Typical multirelational participant network, where the blue node is the original poster, pink nodes are the reposters, and edges still indicate user relationships.

Take the tweet reposting connection shown in Fig. 1(a) and (b) as examples. There are four features in the underlying interaction network: 1) user-D has four followers, B, C, F, and E, but just C and F repost the message created by D; 2) user-F reposts the tweet created by user-D through the intermediary-C; 3) user-C participates more than once; and 4) the tweet created by user-D is also reposted by user-H who does not have the relationship of friend or follower with user-D.

There are two ways of describing these features in previous studies. The conventional method distinguishes whether users interact with each other by adding extra attributes for nodes or edges of declared relationship networks [17], [18], [33]. Fig. 1(c) is the typical application of this method, which attempts to describe Fig. 1(a) and (b) together. However, it cannot depict the features-III and IV. The other solution is to describe all kinds of user relationships or interactions using a multilayer/multirelational network [34], [35]. Likewise, the method cannot express the features-II and III explicitly, and the types of models lack the parallel development of specific analysis methods to exploit the information hidden between the layers.

In response to the above-mentioned problem, we propose a formalized definition of the Weibo information flow (WIF) and have applied it to the empirical analysis of a Sina-Weibo data set on the topic of earthquakes. The goal of our work is to provide a way to extract the underlying hidden social network that more clearly represents the information cascade and actual interactions among users after an earthquake. We will then address the problem in understanding of hidden social networks behind the online information flow.

1) What about the languages used by participants? How is the demographics of participant users? Are there regional features? What are the differences before and after the earthquake?
2) What are the common features of influential users? What are the key factors in user influence?
3) When a user reposts, will he/she evenly repost tweets that attract him/her, or just those tweets posted by his/her friends? Also for users whose message are reposted, does the reposting all take place solely among their followers? Are there any differences in information diffusion patterns before and after the earthquake?
4) Compared with early Web2.0 online communications, does the Sina-Weibo platform have unique features that include social and information dissemination functions?
5) Are there any correlations between the type of interacted user relationships and the topological feature of user networks?

The organization of this paper is as follows. Section III presents the main body of this paper. We first introduce the data set and give a formal definition of the WIF model in Section II. Section III consists of three subsections. Section III-A includes the empirical results of eight individual attributes: the configured language of a personal page, gender, location, the number of followers, the number of friends, the number of posted tweets, the number being reposted, and the correlation among three user rankings. Section III-B analyzes the proportion of each type of user relationship between interacted users and discusses the differences in reposting patterns of each interacted user group by the user relationships. Section III-C uses social network analysis to unveil the topological properties of two types of underlying interaction networks, which are extracted by the WIF model, and analyze the correlation between influential user groups of two networks. Section IV closes this paper with remarks for future work.

## II. Data Set and Methodologies

### A. Data Set

Yi’liang earthquake erupted on September 7, 2012. Ya’an earthquake erupted on April 20, 2013. Because happening consecutively in China, these two natural disasters triggered sparked discussions of Sina-Weibo users. We collected these tweet repost data related two disasters by the Sina-Weibo public application programming interface (API). The API’s registered users can obtain relevant permits as long as that their identities are verified by the Sina-Weibo user authentication. During the course of empirical analyses, we used the MySQL database management system for data extraction and cleansing, and used the Cytoscape toolkit to analyze the network topology [36]. All of the figures are plotted with MATLAB.

As presented in Table I, the data set consists of two parts, demonstrating the contrast between before and after earthquake. Yi’liang county in China was hit by a 5.7-magnitude earthquake on September 7, 2012; the section titled *After Yi’liang Earthquake* is the seismic information generated by Sina-Weibo for seven months after the earthquake. In addition, the section *Before Ya’an Earthquake* is the daily information exchanged on Sina-Weibo regarding Ya’an within a week before April 20, 2013, a 7.0-magnitude earthquake occurred. The object of comparison should be the “*before Yi’liang earthquake*,” but the amount of relevant data is too small for empirical analysis.

**TABLE I:** Sina-Weibo Data Set

Social context of the Dataset	${{NUM}}_{OT}$	${{NUM}}_{RT}$	${{NUM}}_{WU}$	${{NUM}}_{TRT}$	Time span
After Yi’liang Earthquake	9396	407584	330466	3096	Sep 7, 2012 – Apr 30, 2013
Before Ya’an Earthquake	1918	49416	46682	811	Apr 13, 2013 – Apr 19, 2013

${{NUM}}_{OT}$: The count of original tweets; ${{NUM}}_{RT}$: The count of retweets; ${{NUM}}_{WU}$: The count of Sina-Weibo users; ${{NUM}}_{TRT}$: The count of tweet report trees (A tweet repost tree consists of one unique original tweet, it’s retweets and repost relationships among them.).

**Fig. 2.**
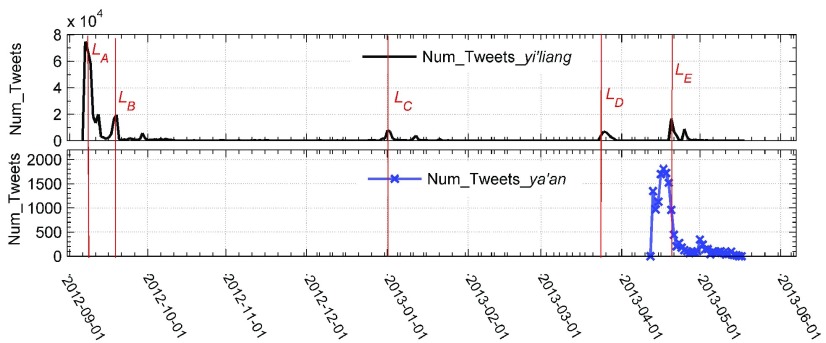
Change in the total number of related original tweets and their retweets over time. Num_Tweets_Yi’liang: number of tweets created after the Yi’liang earthquake, Num_Tweets_Ya’an: number of tweets created before the Ya’an earthquake. Five vertical red lines titled $\text{L}_{A}$, $\text{L}_{B}$, $\text{L}_{C}$, $\text{L}_{D}$, and $\text{L}_{E}$ represent the five dates when important events took place.

Each original tweet record consists of five parts: the original tweet, the original user, repost tweets and their corresponding users, the repost tree, and the relationship between users. Fig. 2 shows the variation trend over time of the total number of these tweets and retweets and five high peaks in the after Yi’liang earthquake data set. These peaks may be related to some sensitive events taking place during those periods. The following social events might offer some available clues.

1) *${L}_{A}$-September 9, 2012:* A 5.7-magnitude earthquake occurred in Yiliang county of China.
2) *${L}_{B}$-September 19, 2012:* The Yiliang county published the latest donations list for Yiliang earthquake.
3) *${L}_{C}$-December 30, 2012:* Called the kindest substitute teacher, Zhu Yinquan accidentally fell from a building, which resulted in severe brain injury, and he had saved seven students by hand in Yiliang earthquake.
4) *${L}_{D}$-March 24, 2013:* The news of relief fund was not distributed in time flashed on the Internet.
5) *${L}_{E}$-April 20, 2013:* A 7.0-magnitude earthquake occurred in Yaan of Sichuan province, China.

### B. Weibo Information Flow (WIF) Model

To describe and retrieve a Weibo-spread information precisely, a formal definition of WIF is proposed as the following quadruple:

\begin{equation*} {{\boldsymbol {WIF}}}={\langle WUS, TS, RRS, TRTS{\rangle }} \end{equation*}

where ***WUS*** is the Weibo user set consisting of participant Weibo users, ***TS*** is the tweet set consisting of original tweets and retweets, ***RRS*** is the repost relationship set consisting of the citing relationships between tweets, and ***TRTS***is the tweet reposts tree set consisting of repost trees of each original tweet.

#### 1) Structure of $\textbf {WU}({\in }\boldsymbol {WUS})$:

\begin{equation*} \textbf {WU}=\langle \textit {gender, pNum, lang, followerSet, friendSet, sCount}{\rangle } \end{equation*}

where *gender* = 0/1 is the “0” to male and “1” to female. *pNum* ${\in }$ *ProvincesNumList*, *PNum* denotes the city where the current user lives (see the Sina-Weibo city code list). *lang* ${\in }$ *{zh-cn, zh-tw, zh-hk, en}* represents the language that the current user has configured on his Weibo page, where “*zh-cn*” denotes the simplified Chinese characters used in Mainland China, “*zh-tw*” and “*zh-hk*” denotes the complex Chinese characters used in Taiwan and Hong Kong, respectively, and “*en*” denotes English. *followerset* is the set consisting of Weibo users who follow the current user. For the *followerSet/friendSet* of each participant, the actual data set mentioned in Table I just records a number, i.e., the number of his/her followers/friends. Of course, for the part of their followers/friends who also participated in the earthquake information dissemination by posting/reposting Sina-Weibo messages, there are detailed records in the data set. *friendSet* is the set consists of Weibo users whom the current user follows. *sCount* is the number of tweets posted by the current user.

#### 2) Structure of $\textbf {T}({\in }\boldsymbol {TS})$ :

\begin{equation*} \textbf {T}=\langle \textit {flag, user, repostCount, time, text, ot}{\rangle } \end{equation*}

where *flag* = 0/1, “0” indicates that the current tweet is an original tweet and “1” indicates responding to the retweet, *user* ${\in }$ *WUS* is the user who posted the current tweet, *repostCount* ${\in }$ *N* is the number of times of the current tweet has been reposted, *time* is the time when the current tweet is posted, *text* is the content body of the current tweet, and *ot* ${\in }$ *TS*, when *flag* = 1, it denotes the original tweet of the current tweet; otherwise, it is null.

#### 3) Structure of $\boldsymbol {RR}( {\in } \boldsymbol {RRS})$:

**Fig. 3.**
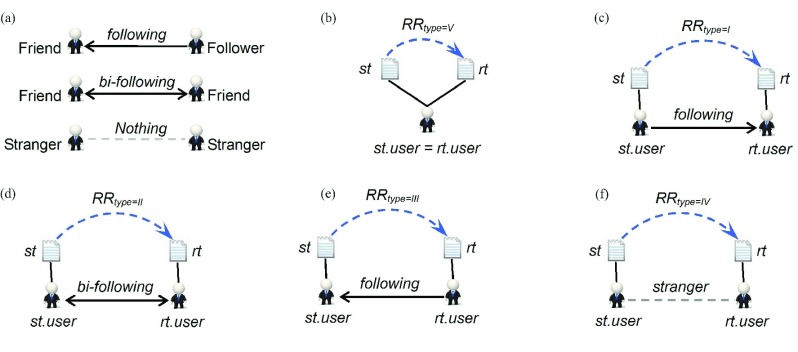
Three types of user relationships and five types of information cascade patterns in WIF. (a) Three types of Weibo_user relationships. (b) *RR* where *type* = V. (c) *RR* where *type* = I. (d) *RR* where *type* = II. (e) *RR* where *type* = III. (f) *RR* where *type* = IV.

\begin{equation*} {\boldsymbol {RR}}=\langle \textit {st, rt, depth, type}{\rangle } \end{equation*}

where $st{\in \{T\mid (T\in TS)\wedge (TrepostCount>0)}\}$ denotes the tweet that was reposted. Regular occurrences of the multilayer reposting phenomenon exist: for example, user-A posts the *ot* first, and then user-B posts the ${rt}_{B}$ by reposting *ot*, and ${rt}_{B}$ are reposted by others, too. Therefore, the *st* is either an original tweet or a retweet. *rt* ${\in \{T\mid (T\in TS)\wedge (T.flag=1)}\}$ is the retweet generated by reposting *st*. *depth* ${\in }$ *N*^+^, indicates the distance between *rt* and *rt.ot* in the repost tree where *N*^+^ = {1, 2, 3, $\ldots $}, and if *RR.st.flag* = 0, then *RR.depth* = 1. *type* ${\in }$ *{I, II, III, IV, V}*, denotes five repost-types by relationship types between the *st.user* and the *rt.user*. Fig. 3(a) shows three types of user relationships among Weibo users where the directed edge indicates that the starting user follows the target user. Here, we refer to the pair of users linked by bidirectional edges as *bi-friend* if they both follow each other, and refer those users as strangers if there are not any directed edges. In this paper, it is assumed that if user-B reposts one tweet posted by user-A, then the tweet information flows from user-A to user-B. After the combination of the information direction between two users and their relationship type, there will be five types of *RRs* [see Fig. 3(b)–(f)]. The following equations are the symbolic descriptions of five types of *RRs*:

\begin{align*}&\hspace {-1.6pc}RRtype \\=&{{V}}\iff RR.st.user=RR.rt.user\\&\hspace {-1.6pc}RR.type \\=&{{I}} \iff ~ (RR.st.user\in RR.rt.user.followerSet) \\&\qquad \qquad \quad \wedge \,(RR.rt.user\notin RR.st.user.followerSet)\\&\hspace {-1.6pc}RR.type \\=&{{II}}\iff ~ (RR.st.user\in RR.rt.user.followerSet) \\&\qquad \qquad \qquad \wedge \, (RR.rt.user\in RR.st.user.followerSet)\\&\hspace {-1.6pc}RR.type \\=&{{III}} \iff ~ (RR.st.user\notin RR.rt.user.followerSet) \\&\qquad \qquad \qquad \wedge \, (RR.rt.user\in RR.st.user.followerSet)\\&\hspace {-1.6pc}RR.type \\=&{{IV}}\iff ~ (RR.st.user\notin RR.rt.user.followerSet)\\&\qquad \qquad \qquad \wedge \,(RR.rt.user\notin RR.st.user.followerSet). \end{align*}

#### 4) Structure of $\boldsymbol {TRT}({\in }\boldsymbol {TRTS})$:

\begin{equation*} {\boldsymbol {TRT}}=\langle {OT, RTS, RRS^{*}, size}{\rangle } \end{equation*}

where *OT* ${\in \{T\mid (T\in TS)\wedge (T.flag=0)}\}$ indicates the original tweet represented by the root node of the current tweet repost tree. *RTS* $^{*}\subseteq \{T\mid (T\in TS)\wedge (T.flag=0)\wedge \,\,(T.ot=OT)\}$ indicates the node set of the current *TRT*, representing *RTs* generated by reposting *OT*. ${RRS}^{*}{\subseteq \{RR\mid (RR\in RRS)\wedge (RR.RT\in RRS^{*})}\}$ indicates the edge set of the current *TRT*, representing *RRs* generated by reposting *OT*. *size* = (*OT.repostCount* +1) indicates the number of nodes of the current *TRT*.

## III. Results and Discussion

### A. Analysis of Users’ Characteristics

This subsection focuses on the following questions. What about the distribution of users’ language? How is demographics of participant users? Are there some regional features? What are the differences before and after the earthquake? What are the typical features of influential users?

#### 1) Basic Analysis:

The demographics, language, and geographic distribution of online users are important measurement indicators of application fields in disaster relief and disease surveillance and control [21], [37], [38]. On Sina-Weibo, the growth spurt of seismic topic tweets emerged as soon as the earthquake occurred. Where did these active users come from? What are their social backgrounds? Our measurement of *WU.gender, WU.lang*, and *WU.pNum* shows: 1) there is a roughly equal proportion of males and females among participant users; 2) users living in the Chinese Mainland, Hong Kong, Macao, and Taiwan are the force that made the tweet peak instantaneously, and the reason of the phenomena mainly come from the locality of Sina-Weibo; and 3) there are a few foreign users participating to post or repost related tweets, but nearly all of them live in China.

**Fig. 4.**
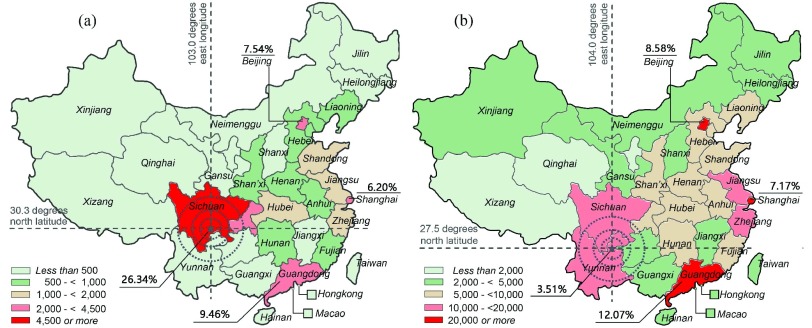
Geographic distributions of Sina-Weibo participants before and after earthquakes in China by province. Geographic distribution of participants (a) before Ya’an earthquake and (b) after Yi’liang earthquake.

Fig. 4 shows the geographic distribution of participant users before and after the earthquake. During the aftermath in Yi’liang which was hit by a 5.7-magnitude earthquake on September 7, 2012, Fig. 4(b) shows that Beijing, Shanghai, and Guangdong topped the rank in the number of participant users and the proportion of active users located in these three areas is 27.83%, these three cities are the political center, the financial center, and the largest province in the economy, respectively. Only 3.5% of active users are located in the local province (Yunnan). In cases prior to the earthquake, there is too little data to analyze unfortunately, probably because Yi’liang is a very remote country in Yunnan province. Therefore, we replaced it with Ya’an which is a popular tourist destination (in the same way later). Then, we found in Fig. 4(a) hat local province (Sichuan) ranked first in the number of active users posting Ya’an common topics, with a share of 26.33%, at the same time the sum of shares in Beijing, Shanghai, and Guangdong still reaches 29.25%.

**Fig. 5.**
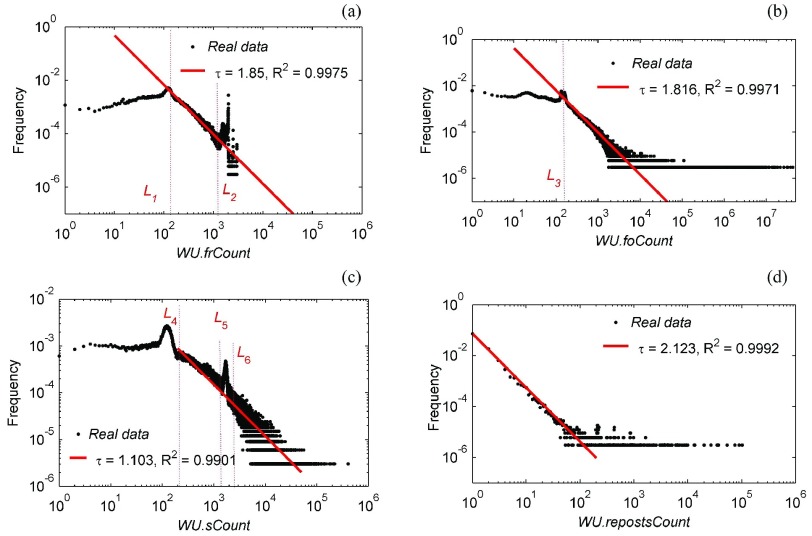
Distributions of four individual attributes of participant users after the Yi’liang earthquake. (a) Number of each participant’s friends, and the fitting interval is located [130, 1300] between $L _{1}$ and $L _{2}$. (b) Number of each participant’s followers, and the fitting interval is located [130,$+ {\infty }$) on the right side of $L _{3}$. (c) and (d) Number of each participant’s friends who also participated in the earthquake information dissemination by posting/reposting Sina-Weibo messages. In addition, “${\tau }$” is the slope of fitting line, “$R ^{2}$” is the coefficient of determination of the fitting procedure.

For participants after the Yi’liang earthquake, Fig. 5 shows the distributions of their four individual attributes the number of friends (*WU.foCount*), the number of followers (*WU.frCount*), and the number of followers/friends who also participated in the earthquake information dissemination by posting/reposting Sina-Weibo (*WU.sCount-P* and *WU.repostsCount-P*), which are counted by the following equations:

\begin{align*} WU.frCount(wu)=&\arrowvert \{wu.friendSet\}\arrowvert \\[2pt] WU.frCount(wu)=&\arrowvert \{wu.followerSet\}\arrowvert \\[2pt] WU.frCount-p(wu)=&\arrowvert \{u\vert u\in wu.friendSet\notag \\[2pt]&\quad \text {and} \quad u\in WUS\}\arrowvert \\[2pt] WU.frCount-p(wu)=&\arrowvert \{u\vert u\in wu.followerSet\notag \\&\quad \text {and} \quad u\in WUS\}\arrowvert. \end{align*}

The fitting function of red fitting lines is the general power model– *frequency* $({X} = {x})= {c}\,\,{\cdot } x^{-z}$ with 95% confidence bounds, where *c,* ${\tau \in R^{+}}$, and $x\in N^{+}$.

Fig. 5 shows the distributions of four individual attributes of participant users. Sudden spikes in Fig. 5(a)–(c) are very obvious. However, we thought that these phenomena have nothing to do with social networks, but are led by the constraints of users privileges on Sina-Weibo. Just with the upper limit of *WU.frCount*, for example, there are three levels: 2000, 2500, and 3000 for nonmembers, ordinary members, and VIP members, respectively (see http://vip.weibo.com/privilege).

Another common feature found in Fig. 5 is segment characteristics. This is true in Fig. 5(a) and (b), where the sudden changes near 130 verify Dunbar’s number. That is, human communities are much larger than those of other primates and hence require more time to be devoted to social maintenance activities. However, there is an upper limit on the amount of time that can be dedicated to social demands, so this sets an upper limit on social group size [41], [42]. It is known that humans have the cognitive capacity to maintain about 150 stable social relationships. With the advent of different types of super social networking services developing one after another, people have once again picked up the topic for discussion [43], [44]. Many researchers have investigated how tools, such as Facebook and Twitter, have changed our capacity to handle social connections using the empirical study [45]–[47]. Here, Fig. 5(a) and (b) also shows the shadow of Dunbar’s number on Sina-Weibo.

In addition, compared with the distribution of broadly defined “friends/followers” which are influenced by the Sina-Weibo user privilege in Fig. 5(a) and (b), the number of each participant’s “actual friends/followers” in Fig. 5(c) and (d) shows the typical power-law distribution; from this, we could know that the unevenness is a universal phenomenon of social networks, even when there are some limiting conditions in social network services, such as the constraint of users’ privileges on Sina-Weibo as described earlier.

#### 2) User Influence:

The number of relevant tweets is the most immediate indication of the popularity of posts on the Weibo space. The number of being reposted can offer us an intuitive view of the original poster’s influence on public opinion. For the popularity, the engagement/productivity, and the influence [5], [40], [48], three types of user rankings are made by the following $R_{T}$, $R_{F}$, and $R_{RT}$

\begin{align*} R_{F}(wu\vert wu\in WUS)=&\vert wu.friendSet\vert \\ R_{F}(wu\vert wu\in WUS)=&\arrowvert \{T\vert T\in TS \wedge T.user=wu\}\arrowvert \\ R_{RT}(wu\vert wu\in WUS)=&\sum _{T\in TS}(T.repostCount \vert T.user\!=\!wu).\notag \\ {}\end{align*}

For the top 20 users of each ranking, the comparison shows that entertainers belong to the most popular group and most users who post tweets actively come from the grassroots, but nearly all users whose tweets regularly receive widespread reposting are news/charity organizations or entertainers. The rest of this subsection quantifies the correlations between the three rankings by the generalized${-\tau }$ model [49]:

\begin{equation*} K_{\tau }^{(0)}(R_{1},R_{2})=\sum _{\{r_{1},r_{2}\}\in (R_{1}\cup R_{2})}\overline {K}_{r_{1},r_{2}}(R_{1},R_{2}) \end{equation*}

where $R_{1}$ and $R_{2}$ are two ordered rankings with the equal length, $k$; and $\overline {K}_{r_{1},r_{2}}(R_{1},R_{2})=1$, if 1) $r_{1}, r_{2}\notin R_{1}\cap R_{2}$ and $r_{1}$ is only in one ranking and $r_{2}$ is in the other ranking; 2) $r_{1}$ is ranked higher than $r_{2}$ and only $r_{2}$ appears in the other ranking; or 3) $r_{1}$ and $r_{2}$ are in both rankings in the opposite order, otherwise, $\overline {K}_{r_{1},r_{2}}(R_{1},R_{2})=0$. In particular, $\overline {K}_{r_{1},r_{2}}(R_{1},R_{2})=\overline {K}_{r_{2},r_{1}}(R_{1},R_{2})$. In addition, the normalized distance-$K$ is used, computed as follows [50]:

\begin{align*} K(R_{1},R_{2})=1-\frac {K_{\tau }^{(0)}(R_{1},R_{2})}{k^{2}},\quad \text {where}~ k=\vert R_{1}\vert =\vert R_{2}\vert.\notag \\ {}\end{align*}

The range of ${K}(R_{1}, R_{2})$ is from 0 to 1. ${K}(R_{1}, R_{2}) = 0$ means complete disagreement, and ${K}(R_{1}, R_{2}) = 1$ means complete agreement.

**Fig. 6.**
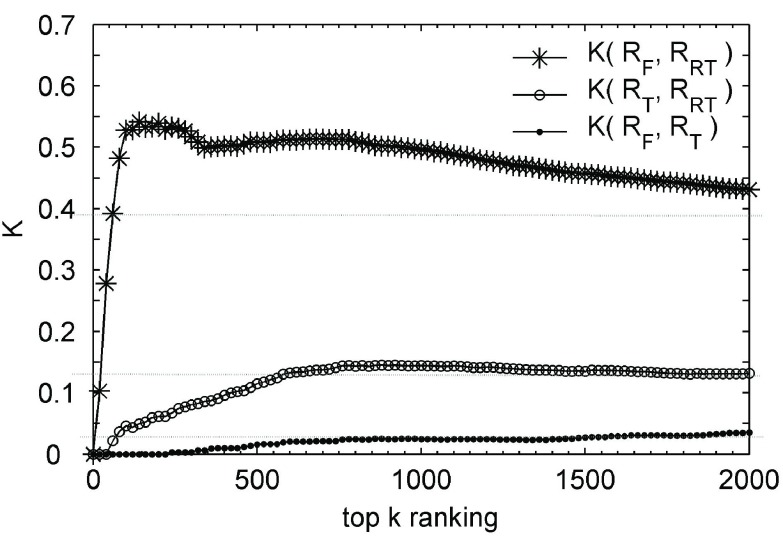
Comparison between three types of user rankings for all users by $R_{F}$, $R_{RT}$, and $R_{T}$.

Fig. 6 shows that with the increase of ${k}$, the distance between two arbitrary rankings will get to a relatively stable level, but three stable levels vary widely. 1) The top association between $R_{F}$ and $R_{RT}$ allow us to infer that a huge fan base may indeed be transformed into a real influence on the public. 2) The weak but stable association between $R_{T}$ and $R_{RT}$ let us deduce that active participation may indeed help to enhance user influence, but it is very limited. 3) There is little or no association between $R_{T}$ and $R_{F}$, so it can be inferred that active grassroots participators seldom intersect with celebrities. Therefore, it can be seen that Weibo is a place shared by the general public and celebrities, which makes Weibo an excellent example of grassroots media. The seldom intersection makes the metamorphosis from grassroots activist to celebrity difficult, but it is a truth that intimate contact can make overnight success possible, though we have not found the hidden principles among this [51].

### B. User Relationship Structure Measurement

**Fig. 7.**
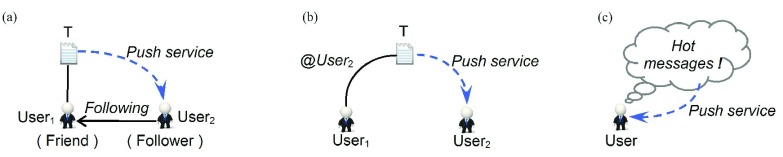
Three main tweet push services on Sina-Weibo. (a) For any two users, User_1_ and User_2_, if User_2_ follows User_1_, then User_1_ is the friend of User_2_ and User_2_ is the follower of User_1_, in addition, tweets posted by User_1_ would be shown for User_2_ in real time. (b) All tweets beginning with the string of “@User_2_” will be shown for User_2_. (c) Every user can read “hot messages” that Sina-Weibo picked out from all tweets of a certain period by their popularity.

From a sociological perspective, friendship is a trust relationship built by repeated games, and now the quality of online social relationships has been receiving increased attention [30], [31], [52]. In our earthquake discussion case, Fig. 7 lists the three main information push services in Sina-Weibo. This section focuses on the following questions. Who actually repost tweets that the original user has posted? When a user reposts, does he/she repost tweets that he/she is interested in, or just those tweets posted by his/her friends? Also for users who get reposted, do the reposting all take place just among their followers? Are there any differences in information diffusion patterns before and after the earthquake?

#### 1) Structure of Weibo Information Flow:

**Fig. 8.**
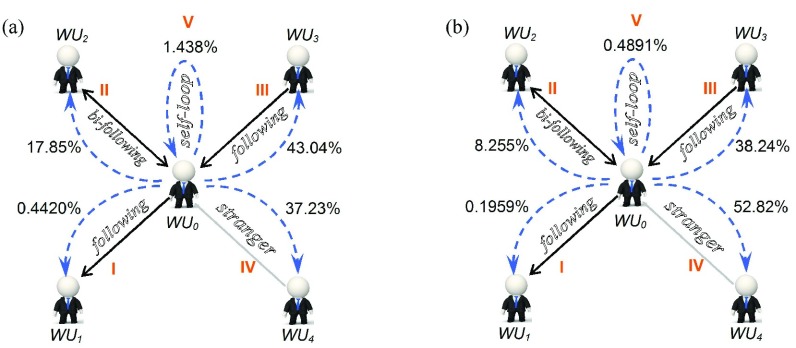
Structure of Sina-Weibo information spread before and after the earthquake, respectively. Notes: five parts marked with ${\vert \thicksim }$IV correspond to the five types of *RRs* in Fig. 3, and the percentages are their shares in the total amount of all *RRs*. Structure of Sina-Weibo information spread during (a) week before the Ya’an earthquake and (b) seven months after Yi’liang earthquake.

For the five types of *RR* in Fig. 3, the butterfly shaped cartoon structure in Fig. 8 analyzed their share in the total amount of *RR* before and after earthquake, respectively, computed as follows:

\begin{equation*} {Ratio}({RRS}_{i})=\frac {\vert {RRS}_{i}\vert }{\vert {RRS}\vert }\times 100\% \end{equation*}

where ${RRS}_{i}=\{ RR{\vert } RR{\in } {RRS}{\wedge } RR.type = i \}$, $ i {\in } \{ I, II, III, IV, V\}$.

For the WIF structure before the Ya’an earthquake, Fig. 8(a) shows as follows. First, the $RR_{type={{III}}}$ from *WU*_0_ to *WU*_3_ shares the largest percentage of all *RRs*, 43.04%, which benefits from the push service in Fig. 7(a). Second, there is no declared relationship between *WU*_0_ and *WU*_4_, but the share of $RR_{type={{IV}}}$ between them reaches up to 37.23%, which may be largely due to the push service in Fig. 7(c). Also, it can be seen that compared with the user popularity, the content of tweets is equally important for their great popularity. Third, the strongest declared friendship exists between *WU*_0_ and *WU*_2_, and the share of $RR_{type={{II}}}$ between them is 17.85%, which can be considered the mirror of the contacting pattern among friends in real life. Fourth, the share of $RR_{type={{V}}}$ is 1.438%. Unfortunately, we found no logical explanation for these behaviors. Fifth, the $RR_{type={{I}}}$ from *WU*_0_ to *WU*_1_ shares the smallest percentage of 0.4420%, less than 1%. Most users did not pay attention to tweets posted by their followers at all. It can be noticed that there is not an information push service from followers to friends, but in the real world, there is also a limited number of efficient ways to attract celebrities. So, this is what we can see in the hedged real world just through the lens of online media.

Fig. 8(b) shows the *WIF* structure after the Yi’liang earthquake, and in Table II, we have listed the comparison result of these two butterfly maps. We can see the proportion of $RR_{type={{IV}}}$ from *WU*_0_ to *WU*_4_ rises remarkably, the growth is more than half.

**TABLE II:** Share of Each Type of RRs in WIF Before and After Earthquake

Social context of the DataSet	Multi-Column				
	Type=I	Type=II	Type=III	Type=IV	Type=V
Before Ya’an Earthquake	0.4420%	17.85%	43.04%	37.23%	1.438%
After Yi’liang Earthquake	0.1959%	8.255%	38.05%	52.82%	0.4891%

In this subsection, the following three conclusions can be drawn. First, “3.1 Analysis of user’ characteristics” has already shown that very few Weibo-big-Vs are able to attract wide attention; in other words, an overwhelming majority of Weibo users play the role of followers. From the dramatic contrast between proportions of $RR_{type={{I}}}$ and $RR_{type={{III}}}$, Sina-Weibo seems like a natural platform to worship idols. Second, given the dramatic contrast among the proportions of $RR_{type={{II}}}$ and $RR_{type={{III}}}$ or $RR_{type={{IV}}}$, Weibo users pay more attention to receiving interesting information than making friends. Maybe this is what makes Weibo different from other online social platforms. Third, followers are indeed the major driving force for the Weibo information spread, but at the same time, the reports from strangers are equally crucial. Particularly in some public emergencies, strangers are likely to be in the vanguard of discussing and distributing news in real time. So, from the point of view of information distribution, in addition to the followers’ count of a user, it is also important to examine whether the tweet is selected as a hot message used to push to all other users (see http://hot.weibo.com/).

#### 2) Distributions of Individual Being Reposted Times:

**Fig. 9.**
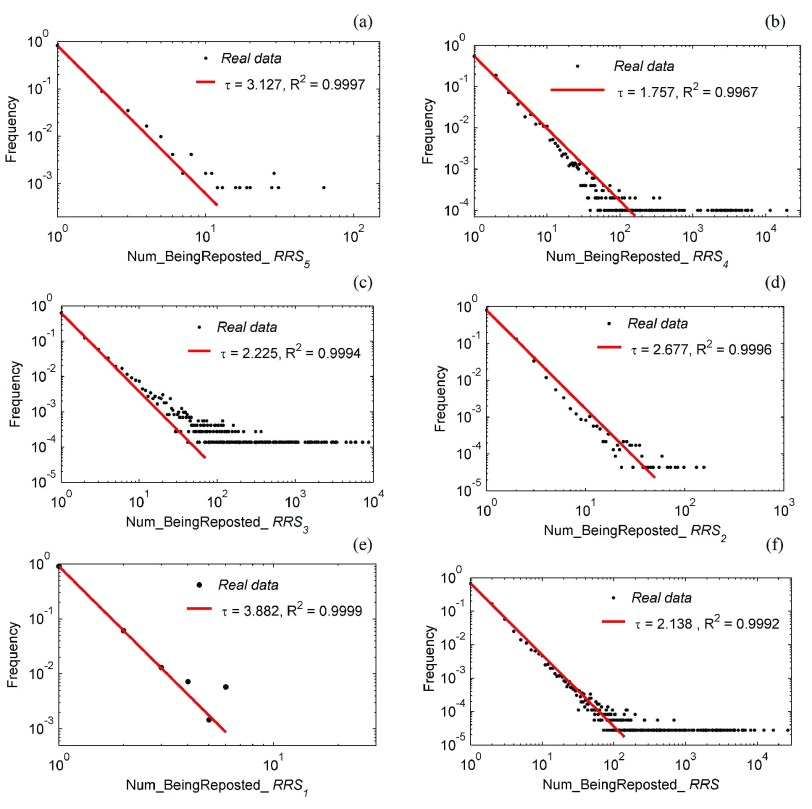
Distributions of the number of being reposted of each *WU* in *RRS* grouped by *RR.type*. (a) RRS of Weibo users’ own reposts. (b) RRS of reposts from strangers. (c) RRS of reposts from followers. (d) RRS of reposts from bifriends (mutually following each other). (e) RRS of reposts from friends. (f) RRS of all reposts.

This subsection presents the distributions of individual reposting times in each type of *RRS* for the *WIF* structure after the Yi’liang earthquake [Fig. 8(b)]. In Fig. 9(f), the statistic sample set is the sum of all *RRs*. If there is a point (${k, p}_{k}$) in the scatter plot, it means that those users whose tweets have been reposted ${k}$ times by others makeup ${p}_{k}$ percentage of participating users total, and there is a following mapping between ${k}$ and ${p}_{k}$:

\begin{equation*} P_{k}=\frac {\sum _{wu\in {WUS}}{count}({wu})}{\vert \{{wu}\vert \exists {RR}({RR}\in {RRS}\wedge {RR}.{st}.{user}={wu})\}\vert }\qquad \end{equation*}

where if the following equation is true, then *count(wu)* = 1, otherwise *count(wu)* = 0:

\begin{equation*} \vert \{RR\vert RR\in RRS\wedge RR.st.user=wu\}\vert =k.\qquad \end{equation*}

The five statistic sample sets in Fig. 9(a) and (e) are ${RRS}_{{{V}}}$, ${RRS}_{{{IV}}}$, ${RRS}_{{{III}}}$, ${RRS}_{{{II}}}$, and ${RRS}_{{{I}}}$, respectively. So, the point ($k$, $p _{k}$) in these five scatter plots means that there is a following mapping between ${k}$ and $p _{k}\!:$

\begin{equation*} P_{k}=\frac {\sum _{wu\in WUS}count(wu)}{\vert \{wu\vert \exists RR(RR\in RRS_{i}\wedge RR.st.user=wu)\}\vert }\qquad \end{equation*}

where *i* ${\in }\{{{I}},{{II}},{{III}}, {{IV}},{{V}}\}$, and if the following equation is true, then *count(wu)* = 1, otherwise *count(wu)* $= 0\!:$

\begin{equation*} \vert \{{RR}\vert {RR}\in {RRS}_{i}\wedge {RR}.{st}.{user}={wu}\}\vert =k. \end{equation*}

All six distributions in Fig. 9 follow a power-law distribution. This is consistent with the distributions of the number of being cited as well as citing others in an empirical study of HFS^6^. However, the three similar power-law slope values (${\tau } =1.68$, 1.75, and 1.84) in the HFS study are equivalent to one distribution (${\tau } =1.757$) of $RR_{type={{IV}}}$, far below those (${\tau } =3.127$, 2.225, 2.677, 3.882, and 2.138) other five distributions. The nature underlying power-law distribution is uneven, and the higher a slope value is, the more severe the imbalance gets. Corresponding to the situation here, it means that these users participating $RR_{type={{IV}}}$ maintain the same interaction pattern with users of ordinary online social forums. However, there might be a serious structural imbalance in the interactions of $RR_{type={{I}}}$, $RR_{type={{II}}}$, $RR_{type={{III}}}$, and $RR_{type={{V}}}$, which may be the reason why Weibo is so different, churning out a string of “Weibo-big-Vs”-Weibo users with mass followings and whose identities have been verified by Sina. These features have to do with social role partitioning [Fig. 3(a)] and the role-based push services (Fig. 7) on Sina-Weibo.

### C. Topology Measurement of Real Interaction Networks

Research by Huberman *et al.* [28] found that the driver of Twitter usage is a sparse and hidden network of connections underlying the “declared” set of friends and followers [53], that is to say in terms of the network density, there is a gap between the real interaction network and the declared user network. Additionally, some other researchers have also found that social interaction existed among various types of users, far more than among acquaintances [54]. From the perspective of network topology, beyond the network density, are there any differences in other topological properties? In addition, what are the key factors behind user influence? To answer these questions, first, a method is introduced to extract the hidden interaction network from the friends and followers network on the basis of the WIF model. Afterward, we present measurement results and provide the corresponding explanation.

**Fig. 10.**
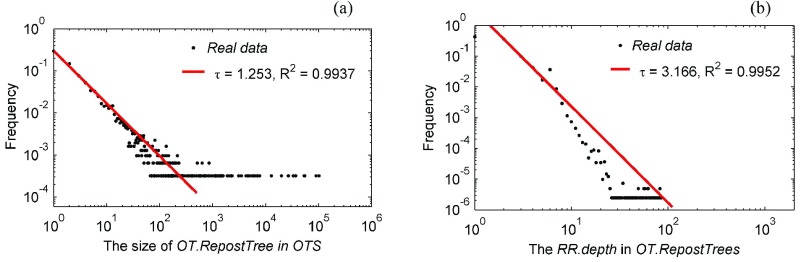
Distribution of *TRT.size* and *RR.depth* in the *WIF* related to the Yi’liang earthquake. (a) *TRT.size* distribution. (b) *RR.depth* distribution.

The *TRT* structure is the key to extracting real interaction networks. For 3096 *TRTS* in the *WIF* after the Yi’liang earthquake, Fig. 10(a) shows their size distribution where the largest *TRT* consists of 102 526 retweets, and Fig. 10(b) shows the *depth* distribution of all *RRs*, where the maximum value is 85.

#### 1) Extraction Process of Two Types of Real Interaction Networks:

**Fig. 11.**
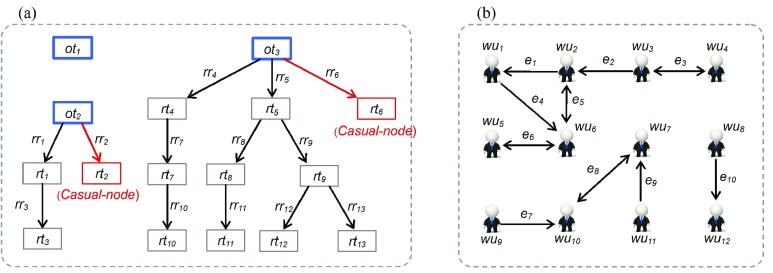
Example of the WIF included 16 *Weibo* messages and 12 *Weibo* users. (a) Structure of the *Tweet Repost Tree Set*, where each node is corresponding to a unique *Weibo* message, and directed edges between pairs of nodes indicate the presence of posting citations between them. (b) Structure of the following-based *Weibo* user declared social network, where each cartoon guy is corresponding to a unique *Weibo* user, and directed edges between pairs of guys indicate the presence of the following relationship between them.

According to the definition of WIF presented in Section II-B, the symbolic representation of the WIF instance in Fig. 11 is listed as follows:

\begin{equation*} WIF = {\langle } WUS, TS, RRS, TRTS{\rangle } \end{equation*}

where *WUS* $= \{wu_{2}$, *WU*_3_, *WU*_4_, *WU*_5_, *WU*_6_, *WU*_7_, *WU*_8_, *WU*_9_, *WU*_10_, *WU*_11_, $wu_{12}\}$. *TS* $= \{ot_{1}$, *ot*_2_, *ot*_3_, *ot*_4_, *ot*_5_, *ot*_6_, *ot*_7_, *ot*_8_, *ot*_9_, *ot*_10_, *ot*_11_, *ot*_12_, ${ot}_{13}\}$, and ${ot}_{1}= {\langle }0$, *WU*_5_, 0, -, -, *null* ${\rangle }$, ${ot}_{2}= {\langle }0$, *WU*_9_, 3, -, -, *null* ${\rangle }$, ${ot}_{3}= {\langle }0$, *WU*_1_, 10, -, -, *null* ${\rangle }$, ${rt}_{1}= {\langle }1$, *WU*_10_, 1, -, -, ${ot}_{2}{\rangle }$, ${rt}_{2}= {\langle }1$, *WU*_4_, 0, -, -, ${ot}_{2}{\rangle }$, ${rt}_{3}= {\langle }1$, *WU*_11_, 0, -, -, ${ot}_{2}{\rangle }$, ${rt}_{4}= {\langle }1$, *WU*_2_, 2, -, -, ${ot}_{3}{\rangle }$, ${rt}_{5}= {\langle }1$, *WU*_2_, 5, -, -, ${ot}_{3}{\rangle }$, ${rt}_{6}= {\langle }1$, *WU*_12_, 0, -, -, ${ot}_{3}{\rangle }$, ${rt}_{7}= {\langle }1$, *WU*_3_, 1, -, -, ${ot}_{3}{\rangle }$, ${rt}_{8}= {\langle }1$, *WU*_6_, 1, -, -, ${ot}_{3}{\rangle }$, ${rt}_{9}= {\langle }1$, *WU*_7_, 2, -, -, ${ot}_{3}{\rangle }$, ${rt}_{10}= {\langle }1$, *WU*_7_, 0, -, -, ${ot}_{3}{\rangle }$, ${rt}_{11}= {\langle }1$, *WU*_6_, 0, -, -, ${ot}_{3}{\rangle }$, ${rt}_{12}= {\langle }1$, *WU*_8_, 0, -, -, ${ot}_{3}{\rangle }$, ${rt}_{13}= {\langle }1$, *WU*_3_, 0, -, -, ${ot}_{3}{\rangle }$. ${RRS}= \{rr_{1}$, *rr*_2_, *rr*_3_, *rr*_4_, *rr*_5_, *rr*_6_, *rr*_7_, *rr*_8_, *rr*_9_, *rr*_10_, *rr*_11_, *rr*_12_, ${rr}_{13}\}$, and $RR_{1} = {\langle }$ *ot*_2_, *rt*_1_, 1, $1{\rangle }$, $RR_{2} = {\langle }$ *ot*_2_, *rt*_2_, 1, $4{\rangle }$, $RR_{3} = {\langle }$ *rt*_1_, *rt*_3_, 2, $4{\rangle }$, $RR_{4} = {\langle }$ *ot*_3_, *rt*_4_, 1, $3{\rangle }$, $RR_{5} = {\langle }$ *ot*_3_, *rt*_5_, 1, $3{\rangle }$, $RR_{6} = {\langle }$ *ot*_3_, *rt*_6_, 1, $4{\rangle }$, $RR_{7} = {\langle }$ *rt*_4_, *rt*_7_, 2, $3{\rangle }$, $RR_{8} = {\langle }$ *rt*_5_, *rt*_8_, 2, $2{\rangle }$, $RR_{9} = {\langle }$ *rt*_5_, *rt*_9_, 2, $4{\rangle }$, $RR_{10} = {\langle }$ *rt*_7_, *rt*_10_, 3, $4{\rangle }$, $RR_{11} = {\langle }$ *rt*_8_, *rt*_11_, 3, $5{\rangle }$, $RR_{12} = {\langle }$ *rt*_9_, *rt*_12_, 3, $4{\rangle }$, $RR_{13} = {\langle }$ *rt*_9_, *rt*_13_, 3, $4{\rangle }$. *TRTS* $= \{trt_{1}$, *trt*_2_, ${trt}_{3}\}$, and ${trt}_{1} = {\langle }$ *ot*_1_, *null, null,* $1{\rangle }$, ${trt}_{2} = {\langle }$ *ot*_2_, $\{rt_{1}$, *rt*_2_, ${rt}_{3}\}$, $\{rr_{1}$, *rr*_2_, ${rr}_{3}\}$, $4{\rangle }$, ${trt}_{3} = {\langle }$ *ot*_3_, $\{rt_{4}$, *rt*_5_, *rt*_6_, *rt*_7_, *rt*_8_, *rt*_9_, *rt*_10_, *rt*_11_, *rt*_12_, ${rt}_{13},\}$, $\{rr_{4}$, *rr*_5_, *rr*_6_, *rr*_7_, *rr*_8_, *rr*_9_, *rr*_10_, *rr*_11_, *rr*_12_, ${rr}_{13}\}$, $11{\rangle }$.

The “declared” online social network is still the common empirical object among existing empirical studies, where nodes represent users, and the edge between nodes indicates the declared social relationship [6]–[8], [39], [48]. Given the prevalence of the Internet water army, this paper is only interested in how participants collaborated with each other and their relationship types. In addition, we found

\begin{align*}&\hspace {-0.6pc}\frac {\vert \{{RR}\vert {RR}\!\in \! {RRS}\!\wedge \!{RR}.{depth}=1\!\wedge \! {RR}.{rt}.{repostCount}=0}{\vert {RRS}\vert } \\&\qquad \qquad \qquad \qquad \qquad \qquad \qquad \qquad \qquad \qquad \qquad \qquad =38.85\% \end{align*}

that is, more than a third of retweets in TRTS link only to the original tweet without any citations relating to other retweets, such as *rt*_2_ and *rt*_6_, in Fig. 11(a). We denoted these types of retweets as casual nodes and the corresponding participants as casual participants. Although casual nodes help spread information (the total number of reposted tweets is an important factor for tweet rank), those nodes did not contribute to the actual collaboration activities. Therefore, we excluded casual nodes and analyzed the remaining repost behavior, which involved a total of 249 237 retweets.

**Fig. 12.**
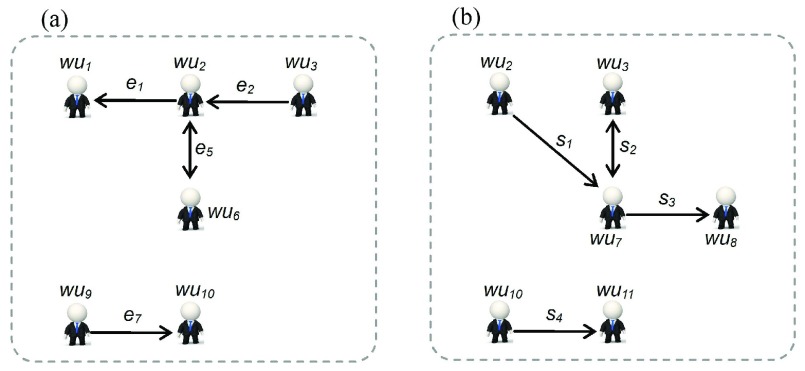
Two types of Weibo message spread participant network. (a) FRCN the FRCN. (b) SRCN the strange reposting cooperation network.

Fig. 12(a) shows one type of real interaction network extracted from the WIF instance in Fig. 11, which was named the friendship-based reposting cooperation network (FRCN). The cartoon figures represent distinct participants, and directed edges indicate the type of social relationships between them [corresponding to the black directed edges Fig. 3(a)]. The extraction process needs to visit all directed edges in Fig. 11(b), retaining only the edges and the participants linked by edges that match the following conditions. That is, if there is a pair of users (${wu}_{i}$, ${wu}_{j}$) linked by edges in FRCN, then in Fig. 11(a) there is at least one *RR* that makes the following formula true:

\begin{align*} \exists {RR}({RR}\in \!{RRS}\wedge \!{RR}.{st}.{user}={wu}_{i}\!\wedge \!{RR}.{rt}.{user}={wu}_{j}).\!\!\!\!\notag \\ {}\end{align*}

Another type of real interaction network is shown in Fig. 12(b); named stranger reposting cooperation network (SRCN) where the cartoon figures represent distinct participants, and directed edges indicate the information flow between them (corresponding the blue directed edges in Fig. 3). The extraction process needs to visit all $RRs$, where type = IV in Fig. 11(b). If there is at least one $RR$ matching the following condition, then we add the corresponding pair of users (${wu}_{k}$, ${wu}_{l}$) and one directed edge from ${wu}_{k}$ to ${wu}_{l}$ into SRCN:

\begin{align*}&\hspace {-0.8pc}\exists RR(RR\in RRS\wedge RR.type=4\wedge RR.ST.user=WU_{k} \\&\qquad \qquad \qquad \qquad \qquad \qquad \qquad \qquad \wedge RR.RT.user=WU_{l}). \end{align*}

Finally, on the basis of the WIF after Yi’liang earthquake, the FRCN consisted of 128 865 nodes and 159 379 edges and the SRCN consisted of 85 978 nodes and 104 410 edges. Therefore, it can be seen again that Sina-Weibo is an excellent synthesis of the traditional society of acquaintances and the strangers’ one.

#### 2) Topology Measurement of FRCN and SRCN:

**Fig. 13.**
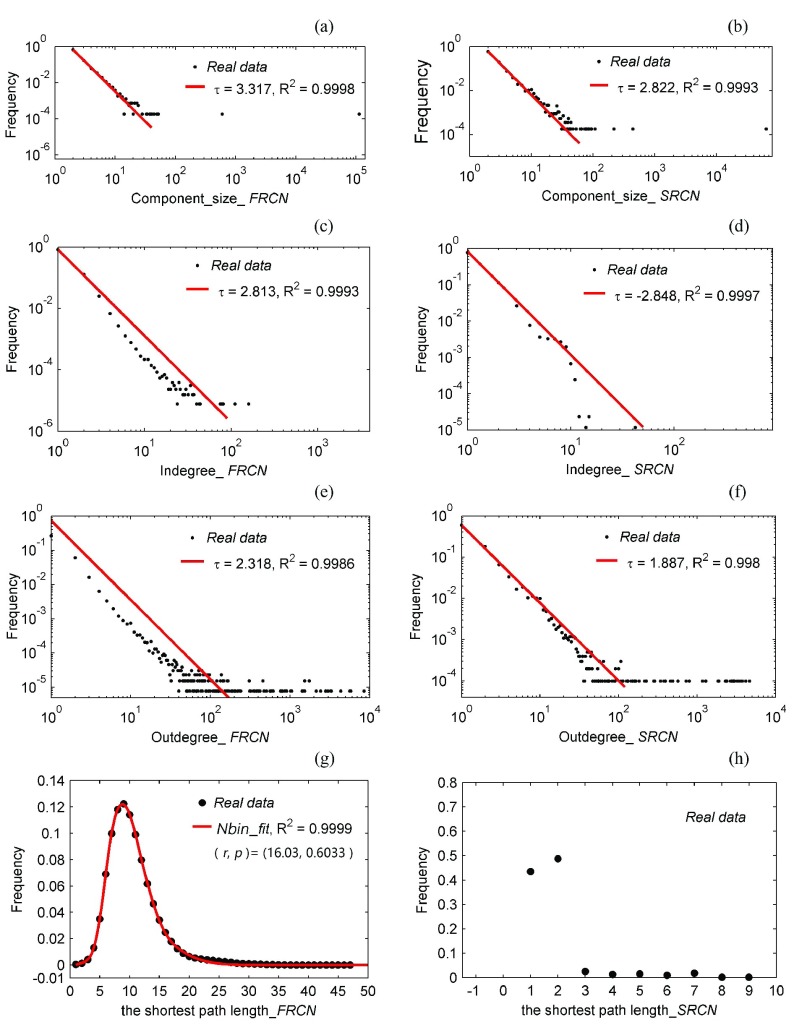
Distributions of three topological properties in FRCN and SRCN. (a) Number of nodes in each component of FRCN. (b) Number of nodes in each component of SRCN. (c) In-degree of each node in FRCN. (d) In-degree of each node in SRCN. (e) Out-degree of each node in FRCN. (f) Out-degree of each node in SRCN. (g) Length of each shortest path in FRCN. (h) Length of each shortest path in SRCN.

Table III lists a comparison of topological properties between FRCN, SRCN, and HFS7; some corresponding distributions are presented in Fig. 13.

**TABLE III:** Topological Properties Comparison of the HFS, the FRCN, and the SRCN

Networks	${N}$	${E}$	${N}_{C}$	${N}_{G}$	${\rho }$	${\tau _{in}}$	${\tau _{out}}$	${C}$	${avg}.{D}$	$S$	$d$
HFS^7^	20 813	29 798	2 821	11 556(55.5%)	10^−4^	2.1	2.4	0.027	2.650	8.679	28
FRCN	128 865	159 379	5 422	112 617(87.4%)	10^−5^	2.813	2.318	0.003	1.998	10.54	47
SRCN	85 978	104 410	104 410	6 241	10^−5^	2.848	1.887	10^−4^	2.401	1.745	9

${N}$: number of nodes; ${E}$: number of edges; ${N}_{C}$: number of components; ${N}_{G}$: number of nodes in the giant component; ${\rho }$: network density; ${\tau _{in}}$: power of in-degree distribution; ${\tau _{out}}$: power of out-degree distribution; ${C}$: average clustering coefficient; ${avg}. {D}$: average degree; ${S}$: average shortest path length; ${d}$: network diameter.

**Fig. 14.**
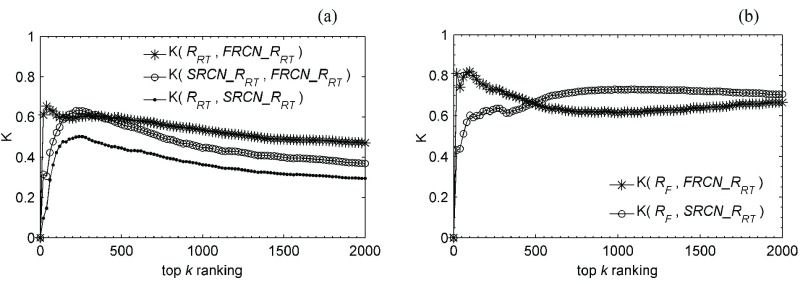
Comparison of user rankings between FRCN and SRCN. (a) Comparison between $R_{RT}$, SRCN_$\text{R}_{RT}$, and FRCN_$\text{R}_{RT}$. (b) Comparison between $R_{F}$, SRCN_$\text{R}{RT}$, and FRCN_$\text{R}_{RT}$.

The values of $\text{N}_{C}$ and $\text{N}_{G}$ in Table III show that there is a largest weekly connected component (giant component) both in FRCN and SRCN, and Figs. 13(a) and 14(b) show that the component size distributions of FRCN and SRCN both follow the power-law trait. Connectivity is an important part of the social network structure, and our analysis results indicate that doing things in groups is a strong human instinct; that is, everything we do or say tends to ripple through our network and have an impact on our friends. For the context of our study, if interest and friendship created the emergence of super components in the HFS groups and FRCN, then what is it that made the emergence of super components in SRCN? Though this question is very interesting, it goes beyond the scope of this paper.

The comparison of ${\rho }$ in Table III shows that values of the network densities of FRCN and SRCN are both much less than the HFS groups; the interaction groups in Sina-Weibo are looser and more disorganized than traditional online social forums, such as HFS communities. This allows Weibo users to group together quickly and leave quickly.

All of the degree distributions in Fig. 13(c) and (f) are the power-law type, meaning that FRCN and SRCN both are scale-free networks. This also means that there are users whose tweets always receive the overwhelming public response (i.e., huge hubs), but the appeal of most of others is severely limited. For the scale-free property in a social network, the research team of Albert-László Barabás holds that it is a consequence of a decision-based queuing process: when individuals execute tasks based on some perceived priority, there will be heavy tail phenomenon [55]. We do not rule out the possibility, because the number being reposted is indeed an important factor for a tweet in a real-time-based push service shown in Fig. 7(c). However, one thing is certain: without the strong appeal of hub users in either FRCN or SRCN, few messages could get widespread.

The comparison of $\boldsymbol {C}$, ***avg.D***, $\boldsymbol {S}$, and $\boldsymbol {d}$ in Table III shows that FRCN and SRCN both are small-world networks. The ***avg.D*** values of FRCN and SRCN are both below the ***avg.D*** of HFS, and their $\boldsymbol {C}$ values are at least an order of magnitude lower than HFS, indicating that most users are not friends of one another. However, the values of $\boldsymbol {S}$ and $\boldsymbol {d}$ in Table III and the result in Fig. 13(g) and (h) both shows that the ***avg.D*** has not affected the social distance, i.e., Weibo information can always flow from one user to another through a small number of hops. With the SRCN, for example, each user has less than three adjacent users on average, but the typical distance between randomly chosen users still remains fewer than nine hops. Another finding about FRCN and SRCN is that the shortest paths of FRCN behave according to the negative binomial distribution.

Finally, on the basis of analysis in Fig. 6, the rest of this section performed further analysis on how much the influential group may converge in FRCN and SRCN with the generalized *Kendal* ${-\tau }$ model again. FRCN and SRCN are two real interaction networks emerging in entirely different ways, so we wondered in particular whether the appeal of these users on the top of $R_{F}$ and $R_{RT}$ maintain the same powerful influence both in FRCN and SRCN. The two formulas in the following are for ranking users of FRCN and SRCN, respectively. And the results are shown in Fig. 14

\begin{align*} FRCN\_{}R_{RT}(wu)=&\vert \{RR\vert RR\in RRS\wedge \neg (RR.type=4) \notag \\&\qquad \qquad \wedge \, RR.st.user=wu\}\vert \\ SRCN\_{}R_{RT}(wu)=&\vert \{RR\vert RR\in RRS\wedge RR.type=4 \notag \\&\qquad \qquad \wedge \, RR.st.user=wu\}\vert. \end{align*}

The total number of times a tweet is reposted is the most concrete indication of user influence. The comparison between the two curves of K($\text{R}_{RT}$, FRCN_$\text{R}_{RT}$) and K($\text{R}_{RT}$, SRCN_$\text{R}_{RT}$) in Fig. 14. A indicates that the influential users in FRCN cater to popular taste better than those in SRCN. The curve of K(SRCN_$\text{R}_{RT}$, FRCN_$\text{R}_{RT}$) in Fig. 14(a) shows that an influential group does converge in FRCN and SRCN, but with limited overlap especially so for the large-scale and widespread reposting. In addition, the two curves in Fig. 14(b) have a similar level, it can be observed that the two influential groups in FRCN and SRCN have at least one thing in common: both have a massive following of fans. Therefore, the determinant of a tweet gets large-scale notice would likely be related to the number of followers the publisher has.

## IV. Conclusion

Weibo has been ubiquitously integrated into people’s everyday lives in China. Both Sina-Weibo and Tencent-Weibo had more than five million users in early May 2013, and their open platforms (including the data API service) have been improved constantly. Although the very existence of user records raises huge concerns of privacy, these user records also create a historic opportunity, that is offering for the first time unbiased data of unparalleled detail on the behavior of not one, but millions of individuals.

In this paper, we performed a comprehensive analysis of Weibo information diffusion during earthquakes. We found that symbolic representation applied to the WIF model is indeed a feasible choice for the empirical study of human behavior based on online social media data sets. In retrospect, our primary inspiration came from the description mechanism of concepts and relationships in ontology theory. The main feature of this idea is that it can give a formal expression for the data structure and the analysis process (such as the extraction of FRCN and SRCN).

However, the structure of social networks is only a starting point. When people talk about the “connectedness” of a social network, in general, they are really talking about two related issues. One is who is linked to whom; and the other is the fact that each individual’s actions have implicit consequences for the outcomes of everyone in the system [56]. In fact, Fig. 8 has given us some intuition that there is probably a serious structural imbalance between the “declared” relationship network and the real interaction network [57]–[59]. In addition, to measure public perceptions in emergencies, many researchers have worked extensively on the evolution of public opinion during information dissemination based on Twitter [60], which allows for many interesting directions for future work.
